# Surgery Averted Using a Novel, Minimally Invasive Approach to Treat Very Severe Radial Artery Spasm

**DOI:** 10.1155/2017/8487056

**Published:** 2017-08-02

**Authors:** Anthony A. Cochet, Daniel A. Bellin

**Affiliations:** Department of Medicine, Cardiology Division, Brooke Army Medical Center, Ft. Sam Houston, TX, USA

## Abstract

A 42-year-old male admitted with a non-ST elevation myocardial infarction was referred for invasive angiographic assessment. Based on preprocedural assessment, the right radial artery approach was selected. Despite possessing none of the consensus risk factors for radial artery spasm, in addition to receiving standard arterial spasm prophylaxis and conscious sedation, the patient suffered very severe radial artery spasm with initial catheter placement, resulting in entrapment of a 5 Fr pigtail catheter within the left ventricle. After exhausting traditional methods for resolution of radial artery spasm, surgical intervention appeared to be the only remaining option for removal of the entrapped catheter. Prior to committing to surgery, use of an axillary nerve block to hinder sympathetic vascular tone was suggested and attempted. This intervention resulted in atraumatic catheter removal. We present a case of very severe radial artery spasm refractory to customary interventions, alleviated with a novel, minimally invasive technique, which spared surgical intervention.

## 1. Introduction

Transradial cardiac catheterization was first introduced as a plausible option for coronary angiography in 1989 by Campeau [1]. Successful percutaneous coronary intervention via radial artery access soon followed as reported by Kiemeneij et al. in 1995 [2]. Use of the transradial approach has become prevalent given its lower risk of bleeding and other vascular complications compared to the more traditional transfemoral approach [3]. Recent studies comparing the safety of radial versus femoral access for percutaneous intervention in ST elevation myocardial infarction have demonstrated a mortality benefit to radial access, decreased risk of major bleeding, and relative risk reduction in bleeding at the access site [4]. The most commonly reported complication in transradial catheterization is radial artery spasm [3]. This case presents a patient referred for invasive coronary angiography who suffered very severe radial artery spasm of the right radial artery, refractory to traditional interventions, requiring regional nerve block of the right axillary nerve for spasm resolution and catheter removal.

## 2. Case Report

The patient referred for invasive coronary angiography was a 42-year-old male, active duty US Army soldier, without significant cardiac history, admitted to Brooke Army Medical Center with chest pain. On the day of admission, the patient noted severe substernal chest pain along with lightheadedness, flushing, and nausea. Symptoms persisted for approximately 45 minutes and resolved spontaneously soon following presentation. Electrocardiogram obtained on arrival revealed diffuse ST segment depressions with an initial serum troponin T reported at 0.02 ng/mL. The patient was hemodynamically stable and free from chest pain at the time of admission. The patient was admitted to the Cardiology service for management of his NSTEMI, with plans to pursue an early invasive strategy. The patient was given aspirin 325 mg orally, clopidogrel 600 mg orally, and atorvastatin 80 mg orally and a continuous heparin infusion was initiated. Troponin T peaked at 0.42 ng/mL twelve hours following admission.

The following morning, the patient was sent for left heart catheterization with coronary angiography with arterial access via the right radial artery. The patient was normotensive and in normal sinus rhythm on arrival to the catheterization laboratory. Midazolam 1 mg and fentanyl 25 mcg were administered intravenously, with appropriate conscious sedation achieved. After standard preparation of the site and subcutaneous infusion of 3 cc of 1% lidocaine, arterial access was obtained without difficulty via modified Seldinger technique and a hydrophilic Terumo 6 Fr Glidesheath Slender was advanced into the vessel followed by administration of intra-arterial verapamil 2.5 mg and nitroglycerin 400 mcg. Continuous intravenous heparin drip was also started prior to initiation of the procedure. A 5 Fr pigtail catheter was advanced into the left ventricle over a J-wire in the standard fashion for evaluation of LVEDP prior to ventriculography.

**Figure 1:**
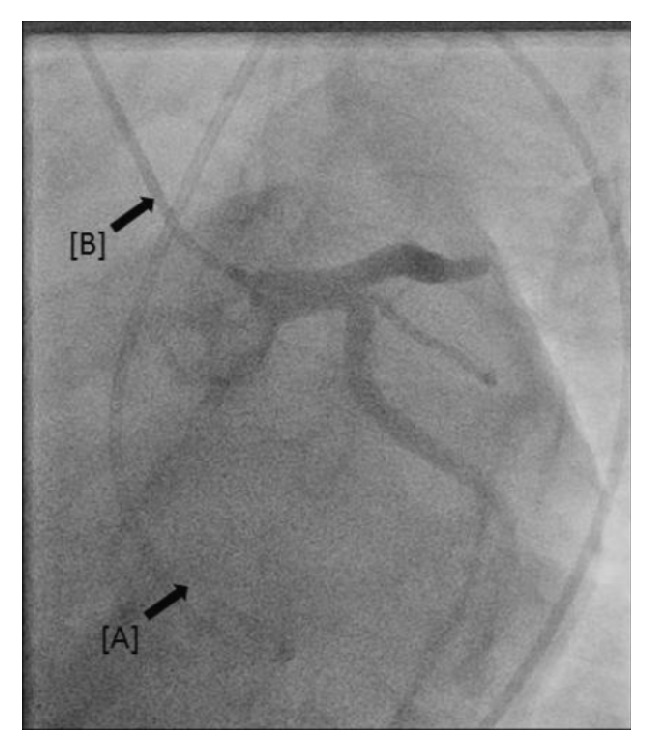
Entrapped 6 French pigtail catheter via right radial access [A]; Judkins Right 6 French catheter engaging the left main coronary artery via the right femoral approach [B].

Immediately following catheter placement, the patient experienced probable acute radial artery spasm, preventing either antegrade or retrograde movement of the catheter. Manipulation of the catheter was discontinued for 5–10 minutes to allow for spontaneous resolution of the spasm; however, catheter entrapment persisted. Additional conscious sedation with fentanyl 50 mcg and midazolam 1 mg was administered intravenously, warm compresses were applied, and supplemental intra-arterial infusions of nitroglycerin and verapamil were provided, again without resolution of catheter entrapment. Escalation of vasodilator therapy was then pursued with initiation of a nicardipine infusion at a rate of 12.5 mg/hour. After sufficient time was expended for medication effect to occur, attempt at catheter removal was again unsuccessful. Local subcutaneous administration of additional lidocaine and nitroglycerin was ineffective as well. Right femoral access was then obtained and the procedure resumed, revealing normal coronary angiography and left ventricular hemodynamics (Figure 1).

With multiple traditional interventions for relief of radial spasm exhausted, consultation with anesthesiology was pursued to assist with escalation of sedation. The patient was converted by the anesthesia team to general sedation, first with propofol infusion followed by a dexmedetomidine infusion. Catheter entrapment nonetheless persisted. At this time, surgical consultation with vascular surgery and cardiothoracic surgery was obtained, as surgical intervention appeared a distinct possibility for catheter removal.

A final attempt at nonsurgical intervention was collectively decided upon, with suggestion made to attempt regional axillary nerve block as a means of reducing sympathetic tone attributing to the severe vasoconstriction. Under ultrasound guidance, 40 cc of 1.5% mepivacaine without epinephrine was infused slowly in the location of the right axillary nerve. Right upper extremity venous engorgement and vasodilation were appreciated on ultrasound during infusion. Approximately seven minutes after completion of the infusion, gentle traction was placed on the radial catheter, resulting in atraumatic removal of both the sheath and catheter.

The patient was monitored as an inpatient for 24 hours. He regained full function of his right arm without notable neurovascular deficiency. He has been followed up routinely as an outpatient without development of such deficiencies.

## 3. Discussion

This case demonstrates very severe radial artery spasm during diagnostic catheterization that exhausted the traditional methods utilized to both prevent and relieve radial artery spasm [5]. Although the transradial approach presents a safe alternative to the transfemoral approach in its reduced instances of major bleeding and other vascular complications, radial artery spasm remains a leading complication of the procedure [3]. Review of the literature reports instances of radial artery spasm of any severity as high as 14.7% to 24% [3, 6]. In a recent review, rates were much lower, at 5.6%, when studied in cases performed by experienced operators [5].

Risk factors for development of radial artery spasm include female gender, large sheath size, multiple catheter exchanges and/or frequent catheter manipulation, anatomic variations of the radial artery, and operator inexperience [6]. A number of proposed methods have been evaluated in the prevention and treatment of radial artery spasm. However, radial artery spasm occurs at varying degrees of severity and no single intervention has proven successful in the resolution of all instances of this complication. The standard accepted method for prevention of spasm includes administration of intra-arterial vasodilators (nitroglycerin and verapamil being most common), use of hydrophilic sheaths, and local anesthesia [3, 6–8]. Conscious sedation and pain control appear to play a role in the prevention of radial artery spasm. However, no consensus yet exists on the routine implementation of preprocedural sedation in transradial catheterization to prevent this complication [8].

Radial artery spasm is categorized based on the degree of pain, limitations of catheter movement, and interventions required to resolve the spasm. The degree of spasm is classified as mild, moderate, severe, or very severe (Table 1) [5]. Multiple proposed methods for resolution exist in the literature and in practice. Initial steps include warm compresses applied to the access site, repeat administration of intra-arterial vasodilators, subcutaneous analgesia/vasodilators, and allowing for adequate time without catheter manipulation for spasm resolution [3, 5, 6, 8]. Radial artery angiography has also been suggested for evaluation of the degree of spasm and overall vascular anatomy. Rarely in severe cases, where conservative therapy has failed to relieve the spasm, increased systemic analgesics and general anesthesia have been required to quell the spasm [8].

The radial artery spasm discussed in this case represents a rare, severe form of the complication, in which established therapy was unsuccessful. In general, the patient lacked many of the traditional risk factors for radial artery spasm. Radial access was obtained quickly, without multiple punctures or difficulty with guidewire passage. A hydrophilic, slender sheath was utilized for radial cannulation. Catheter entrapment occurred immediately following passage of the initial diagnostic catheter, which required no significant manipulation for advancement into the left ventricle. The patient had received intravenous midazolam and fentanyl preprocedurally and he complained of no pain with access or catheter advancement, remaining consciously sedated throughout.

Several unique circumstances existed which confounded the management of this complication. First, attempts at flushing or drawing from the sheath side port directly were unsuccessful and the pigtail catheter tip remained in the left ventricle. Therefore, radial artery angiography was unobtainable through either the sheath or the entrapped catheter, and infusion of spasmolytics through the sheath was prohibited. Second, given the location of the catheter tip within the left ventricle, surgical removal of the catheter may have required utilization of vascular and/or cardiothoracic surgeons. As general anesthesia failed to resolve the spasm, surgical intervention would have been the next intervention pursued had regional nerve block not been attempted.

Although transradial catheterization continues to increase in prevalence worldwide, in the United States, it represents less than 10% of all invasive angiography [9] and less than 2% of percutaneous interventions [3]. As centers continue to increase the volume of transradial catheterizations performed, the prevalence of this complication will be encountered by angiographers with increasing frequency.

## 4. Conclusion

We present a case of very severe radial artery spasm, refractory to traditional methods of resolution, in which surgical intervention for removal of an entrapped catheter was avoided with the use of a regional axillary nerve block—a novel technique that, to our knowledge, has not been published as a proven method to treat this complication. This case also demonstrates that significant radial artery spasm can occur even in a patient lacking many of the significant risk factors associated with this complication.

## Figures and Tables

**Table 1:** Classification of radial artery spasm.

Grade I	Mild spasm	Minimal pain/discomfort along course of RA during/following procedure.
Grade II	Moderate spasm	Significant pain/discomfort along course of RA during/following procedure.
		Catheter manipulation possible to complete procedure.

Grade III	Severe spasm	Severe pain/discomfort along course of RA with catheter movement despite administration of at least 2 spasmolytic cocktails.
		Procedure completion possible with balloon-assisted tracking (BAT) technique or small-diameter catheters.

Grade IV	Very severe spasm	Very severe pain/discomfort along course of RA with catheter entrapment despite administration of at least 2 spasmolytic cocktails.
		Refractory to BAT or other salvage techniques.

Classification/criteria as defined by Patel et al., 2014.
